# Systematic study on G‐protein couple receptor prototypes: did they really evolve from prokaryotic genes?

**DOI:** 10.1049/iet-syb.2013.0037

**Published:** 2014-08-01

**Authors:** Zaichao Zhang, Zhong Jin, Yongbing Zhao, Zhewen Zhang, Rujiao Li, Jingfa Xiao, Jiayan Wu

**Affiliations:** ^1^ CAS Key Laboratory of Genome Sciences and Information Beijing Institute of Genomics, Chinese Academy of Sciences No.1-7, Beichen W Rd, Chaoyang District Beijing People's Republic of China 100101; ^2^ College of Life Science, Graduate University of Chinese Academy of Sciences No.9A Yuquan Rd, Shijingshan District Beijing 100049 People's Republic of China; ^3^ Supercomputing Center Computer Network Information Center, Chinese Academy of Sciences No.4, South Four Street Zhongguancun, Haidian District Beijing 100190 People's Republic of China

**Keywords:** proteins, molecular biophysics, genetics, biochemistry, molecular configurations, bioinformatics, data mining, systematic study, G‐protein couple receptor prototypes, prokaryotic genes, GPCR, signalling proteins, eukaryotes, metabotropic glutamate receptor family, phylogenetic analysis, GABA, bacteriorhodopsin, periplasmic binding protein, chemical union, GPCR orphans prediction, GPCR orphans classification

## Abstract

G‐protein couple receptor (GPCR) is one of the most striking examples of signalling proteins and it is only observed in eukaryotes. Based on various GPCR identification methods and classification systems, several evolutionary presumptions of different GPCR families have been reported. However, the prototype of GPCR still limits our knowledge. By investigating its structure and domain variance, the authors propose that GPCR might be evolved from prokaryotic world. The results given by the authors indicate that metabotropic glutamate receptor family would be the ancestor of GPCR. Phylogenetic analysis hints that one of metabotropic glutamate receptor GABA is possibly formed and evolved from the ancient chemical union of bacteriorhodopsin and periplasmic binding protein. The results obtained by the authors also unprecedentedly demonstrate that specific domains and identical structures are shown in each type of GPCR, which provides unique opportunities for future strategies on GPCR orphans’ prediction and classification.

## 1 Introduction

G‐protein couple receptor (GPCR), also known as 7‐transmembrane domain receptors, is one of striking protein families in cellular signalling mechanism. It represents an essential branch of gene families only shown in eukaryotic kingdom. GPCR has great diversity in sequence alignment with a basal structure framework. It has a tertiary structure that not only resembles a barrel inserted in cellular membrane but also consists of three primary components: extracellular *N* ‐terminus, transmembrane region and intracellular *C* ‐terminus. Transmembrane region contains 7‐transmembranic helixes (TM1 ∼ TM7), three extracellular loops (EL1 ∼ EL3) and three intracellular loops (IL1 ∼ IL3).

Basically, GPCR has three main families as classified in GPCR database (www.gpcr.org/7tm/). Rhodopsin‐like receptor family (class A), consisting of more than 20 subclasses, is the largest GPCR group and also represents in most vertebrate genomes [1]; secretin receptor family (class B) has a small number and it mainly acts as hormone or neuropeptide; metabotropic glutamate receptor family (class C) performs a variety of functions in behavioural and mood regulations and nervous systems. It is also reported that receptors in this family have a specific structure showing some similarities with signal‐related structures in prokaryotic genomes [2]; except the three main families, there are also a few other families such as fungal mating pheromone, frizzled or smoothened and orphan receptors. In this study we only focus on the three major families.

Previous work has provided insights on GPCR evolution focusing on two perspectives: sequence comparisons between different species and populations within one species [3]. As they mainly focused on GPCR evolution from individual family, they failed to mention a brief systematic ancestor of GPCR and the correlation among different families [4–8]. Since metabotropic glutamate receptor family has been found in ancient slime molds and sponges [2], several clues show that the phylogenetic oldest GPCR would be glutamate‐receptor‐like receptors [9–11]. Therefore it is necessary to figure out a possible evolutionary mechanism of GPCR metabotropic glutamate receptor family. The increasing number of complete genome sequences provides us a great opportunity to raise a GPCR evolutionary question: did GPCR evolve from the ancient prokaryotic world?

Here, we preformed comparative analyses of three primary GPCR families and identified the correlation of specific structures of metabotropic glutamate receptor family with prokaryotic proteins. Several identical features of different GPCR listed in this study would provide us better understanding for GPCR orphans’ prediction and classification.

## 2 Materials and methods

### 2.1 Data mining and classification

GPCR sequences were downloaded from GPCRDB (www.gpcr.org/7tm/) and aligned by CD‐HIT at 90% identity [12]. Twenty types of rhodopsin‐like receptor subfamily, 18 types of secretin receptor subfamily and eight types of metabotropic glutamate receptor subfamily were applied in our study (Table 1). periplasmic binding proteins (PBP) and bacteriorhodopsin sequences were taken from NCBI (ftp://ftp.ncbi.nlm.nih.gov/, on June 2012) and trained by NCBI bacterial database using BLAST with E‐value 10^−5^ [13]. The final PBP and bacteriorhodopsin sequences were aligned and filtered by CD‐HIT at 85% identity.

**Table 1:** Domain list and eukaryotic hierarchies of each GPCR subfamilies^a^

Family	Subfamily	Protists and fungi	Vertebrate	7‐transmembrane	*C* ‐terminus domain	*N* ‐terminus domain
*A*	amine	+	+	7tm_1		
*B*	cadherin_EGF_LAG_CELSR	+	+	7tm_2		
*B*	calcitonin	+	+	7tm_2	Hid1,TFIIA	HRM
*C*	calcium_sensing	+	+	7tm_3		ANF_receptor, NCD3G
*A*	cannabinoid	+	+	7tm_1		
*A*	CAPA	−	+	7tm_1		
*B*	CD97	+	+	7tm_2		EGF_CA, GPS
*B*	corticotropin_releasing_factor	−	+	7tm_2		HRM
*B*	depsiphilin	+	+	7tm_2		Gal_Lectin, HRM, DUF3497, GPS
*B*	diuretic_hormone	+	+	7tm_2		HRM
*A*	ecdysis_triggering_hormone	−	+	7tm_1		
*B*	ERM1	+	+	7tm_2		EGF_CA, GPS, DUF3497
*B*	FLG_hepta	−	+	7tm_2		
*C*	GABA_B	+	+	7tm_3	eIF‐4B,Vpu	Sushi, ANF_receptor
*B*	glucagon	−	+	7tm_2		HRM
*A*	gonadotropin_releasing_hormone	+	+	7tm_1	SCA7	
*B*	GPRX	+	+	7tm_2		Somatomedin_B, DUF3497, Lectin_C, I‐set, LRR
*B*	growth_hormone	−	+	7tm_2		HRM
*A*	hormone	+	+	7tm_1	AMH_N	LRR, GnHR_trans
*A*	hydroxycarboxylic_acid	−	+	7tm_1		
*B*	latrophilin	+	+	7tm_2	zf‐SAP30	DUF3497, GPS
*A*	leukotriene_B4_receptor	−	+	7tm_1		
*A*	lysosphingolipid_and_LPA_EDG	−	+	7tm_1		
*A*	melatonin	+	+	7tm_1		Ribosomal_S27e, Rho_N
*C*	metabotropic_glutamate	+	+	7tm_3	DUF2981	ANF_receptor, NCD3G
*B*	methuselah_like_proteins_MTH	+	+	7tm_2	PA14,methuselah_N	Methuselah_N, Birna_VP4
*A*	nucleotide_Like	+	+	7tm_1		
*C*	odorant	+	+	7tm_3		ANF_receptor, NCD3G
*A*	olfa	+	+	7tm_4,7tm_1,7TM_GPCR_Srsx	peptidase_M10, MED14	
*A*	olfactory	+	+	7tm_1,7TM_GPCR_Srw	MED14, metallopep,peptidase_M10	
*A*	orphan	+	+	7tm_3		Ld1_recept_a, LRR, PRKCSH_1
*C*	other	+	+	7tm_2	DUF3667	ANF_receptor, NCD3G
*B*	parathyroid_hormone	−	+	7tm_2		HRM
*B*	PDF	+	+	7tm_1		Myb_DNA‐bind_5, HRM
*A*	peptide/cell surface receptor	+	+	7TM_GPCR_Srw,7tm_4		
*C*	phermone	+	+	7tm_3		ANF_receptor, NCD3G
*A*	platelet_activating_factor	−	+	7tm_1		
*A*	prostanoid	−	+	7tm_1		
*A*	rhod_opsin	+	+	7tm_1		Rhodopsin_N, Pkinase_Try,UBA
*B*	secretin	−	+	7tm_2		HRM
*C*	taste	+	+	7tm_3		ANF_receptor, NCD3G
*A*	thyrotropin_releasing_hormone	+	+	7tm_1		
*B*	to_be_sorted	+	+	7tm_2	NCD3G	ECF_CA, DUF3497, GPS, HRM
*B*	vasoactive_intestinal_polypeptide	−	+	7tm_2		HRM
*A*	viral	−	+	7tm_1		
*C*	vomeronasal	+	+	7tm_3		ANF_receptor, NCD3G

a ‘ + ’ indicates that we have found at least one gene in that level and ‘ − ’ means not. Domains were predicted by Pfam Version 26.0 and related abbreviations refer to Pfam website. Species list is shown in supplementary Table 1.

### 2.2 GPCR transmembrane and domain prediction

*N* ‐terminus, seven‐transmembranic helixes and *C* ‐terminus of all GPCR were predicted by TMHMM server (http://www.cbs.dtu.dk/services/TMHMM/) and we selected 7‐transmembrane helix (about 71%, 1661 out of 2468) for further investigation. The Pfam database (version 26.0, http://pfam.sanger.ac.uk), a large collection of protein families representing by multiple sequence alignments and hidden Markov models, was employed to detect GPCR domains with *E* ‐value 0.1.

### 2.3 Phylogenetic analysis

ClustalW2 was used to align PBP sequences and *N* ‐terminus [14]. Bacteriorhodopsin sequences with seven‐transmembranic helix were predicted by TMHMM and then aligned with GPCR 7‐transmembrane. We used ProtTest2.4 to determine the best fit amino acid substitution model (JTT + F) with parameter values for maximum likelihood analyses [15]. Phylogenetic tree was constructed and visualised by MEGA5 with 1000 of bootstrap replicates [16].

### 2.4 Conservative analysis in eukaryotic kingdom

In our study, we focused on GPCR types existing in organisms that are observed before and after the appearance of metazoan about 550 million years ago. This period has been widely considered as a ‘big bang’ of the bilaterians, animals with a front and a back. Based on the phylogenetic tree of known species (http://tolweb.org/tree/), 17 most representatively complete sequenced and annotated eukaryotic genomes with wide hierarchies ranging from protists to mammals were selected from NCBI. We utilised BLAST and eukaryotic annotation information to see GPCR distributions in each eukaryotic period. We classified fungi and protists as species occurred before the exploration of metazoan and the rest species are vertebrates. A list of species applied in this study can be found in supplementary Table 1.

## 3 Results and discussions

### 3.1 Overall structure and domain distributions

**Fig. 1:**
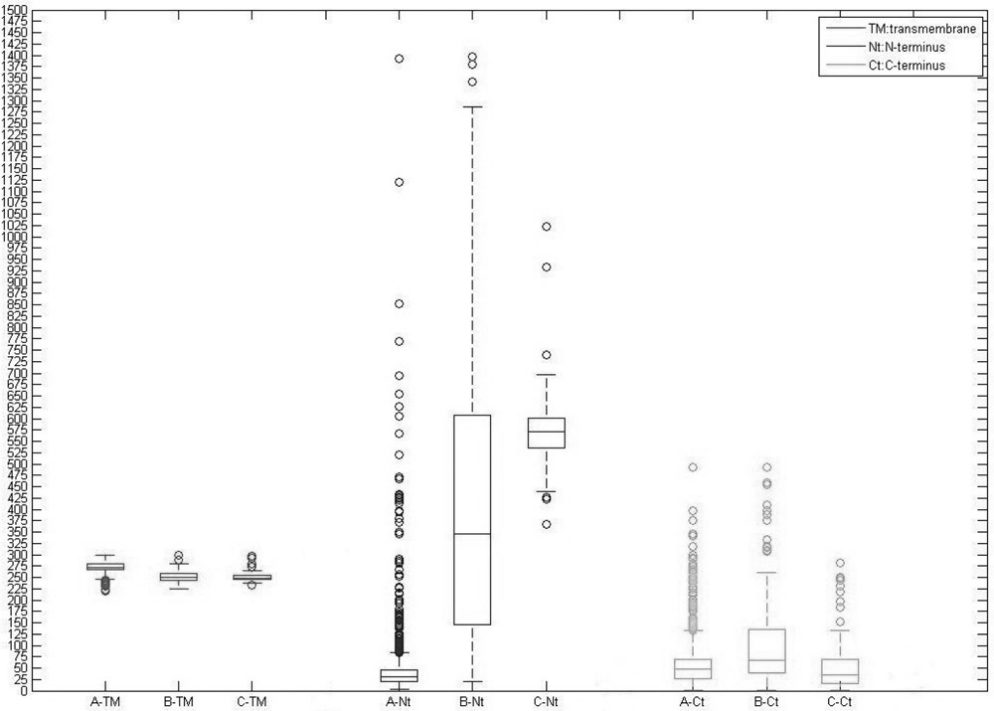
Amino acid length of each part of GPCR (A: Rhodopsin‐like receptor; B: Secretin receptors; C: metabotropic glutamate receptor)

Sequence alignment shows that each part of GPCR has a significant sequence variance. However, 7‐transmembrane structure is considerably conserved with the nucleotide length ranging from 200 to 300 amino acids (Fig. 1). Nucleotide length and specific domain on *N* ‐terminus vary dramatically among different families (Table 1). Most domains detected on TM1 ∼ 7 are 7 tm_n and DUFx (*n* is 1 ∼ 7 and *x* is a four digital number). DUFx composes of a large protein family whose domains are still unknown. Interestingly, EL2, IL3 and EL3 show some differences among three families (supplementary file 1). It is possible that the functions of ligand‐binding sites of EL2 and EL3, which receive signals from extracellular, were reinforced *N* ‐terminus in rhodopsin during rhodopsin‐like receptor evolution.

Many domains on *N* ‐terminus were detected such as epidermal growth factor, cadherin domain and RGD. Some diversified domains are also found in *N* ‐terminus of different GPCR. Taking cysteine residue box for an example, it might mediate cell‐to‐cell adhesion and migration [10, 17–20]. An interesting thing is a great number of *N* ‐terminus on rhodopsin‐like receptors are much shorter than those on the other GPCR families. Short *N* ‐terminus structure promotes extracellular loop to ligand‐binding sites for receiving extracellular signals. We observed *N* ‐terminus from metabotropic glutamate receptor family containing ANF receptor and NCD3G, whereas *N* ‐terminus from secretin receptor has the same HRM domain. Rhodopsin‐like receptor subfamilies only found in metazoan have few domains on *N* ‐terminus. For instance, the hormone receptor from rhodopsin‐like receptor family is detected in both periods (before and after metazoan). This implies these receptors probably are forefather of rhodopsin‐like receptor family. As for *C* ‐terminus, it varies considerably in length and specific domains were hardly found. *C* ‐terminus only contains serine or threonine residues to increase the affinity of intracellular surface for the binding of scaffolding proteins, especially when these residues are phosphorylated [20].

### 3.2 GPCR 7‐transmembrane might evolve from bacteriorhodopsin

Bacteriorhodopsin is an ancient light energy related protein that widely found in prokaryotes. It was reported in 1992 that bacteriorhodopsin might contain a similarity structure with 7‐transmembrane structure [21]. Electron‐crystallographic refinement of bacteriorhodopsin revealed that some analogy existed between GPCR and bacteriorhodopsin [3]. Evidence shows 7‐transmembrane region is conserved in the crystal structure of rhodopsin [22], and it was also identified as the similar structure as in prokaryote genomes like light‐sensitive proteo‐, bacterio‐ and halorhodopsin [23, 24].

**Fig. 2:**
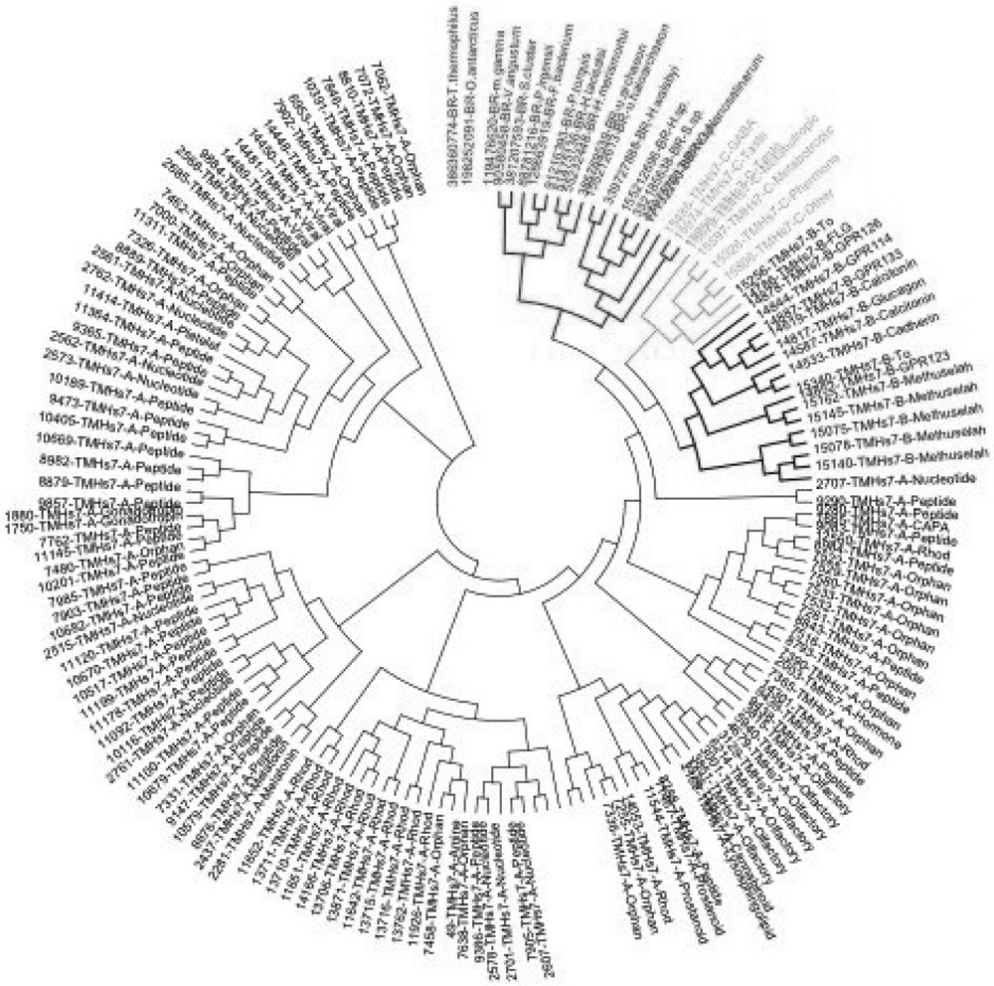
Phylogenetic tree of bacteriorhodopsin and 7‐transmembrane region (Cut‐off value for condensed tree is 50%; Dark Grey: BR Bacteriorhodopsin; Light Grey: C metabotropic glutamate receptor; Bold Black: B Secretin receptors; Black: A Rhodopsin‐like receptor)

Based on these previous assumptions, we searched the entire bacteria database from NCBI and the most hits are bacteriorhodopsin. We found 830 out of 1096 trained bacteriorhodopsin sequences with a length of 200 ∼ 300 amino acids consisting of 7‐transmembrane region. To further investigate their phylogeny, we constructed a phylogenetic tree by the sequences from all GPCR *N* ‐terminus and bacteriorhodopsin by maximum likelihood method with bootstrap replicates of 1000. Because there is great variance between GPCR *N* ‐terminus, the bootstrap value decreases rapidly in the *N* ‐terminus branch. Therefore we only focused on the structure of this phylogenetic tree. It shows that 7‐transmembrane of GPCR metabotropic glutamate receptor family has a much closer relationship with bacteriorhodopsin than other GPCR families (Fig. 2). GABA, taste, metabotropic and pheromone from GPCR metabotropic glutamate receptor subfamilies would be more ancient than secretin receptors and rhodopsin‐like receptors.

We also observe that 7‐transmembrane region contains polarity conserved positions, which also have been reported in bacteriorhodopsin [25]. Metabotropic glutamate receptor family has few introns whereas rhodopsin‐like receptor family contain 35.5% introns and the introns of secretin receptor family are highly conserved in their position [26]. The popular consensus at present is that introns appear within the eukaryote lineage as selfish elements, which proves metabotropic glutamate receptor family is the possible ancestor of GPCR. The possible reason is fewer introns would be propitious for gene duplication under evolutionary pressure. Some other domains (e.g. Bac_rhodopsin, AtpR, Sugar_transport, T2SM) also present on bacteriorhodopsin [27–33]. Since bacteriorhodopsin has more diversified domains, it implies bacteriorhodopsin may play other functions in prokaryotic life activities as well. The issue that whether sequence homology between bacteriorhodopsin and GPCR is formed by exons shuffling or duplication is still controversial [34], but obviously we could draw a conclusion that the origin of 7‐transmembrane region is possibly from bacteriorhodopsin in the prokaryotic world.

### 3.3 PBP might be the prototype of GPCR N‐terminus

Previous studies have reported that metabotropic glutamate receptor *N* ‐terminus and the ancient PBP share the identical structure Venus flytrap module (VFTM). VFTM has been suffered three rounds of duplication with positively selected functional divergence [17, 35]. Conformational changes of VFTM induced by ligand‐binding sites might have a correlation with the prototype of *N* ‐terminus.

Studies on the metabotropic glutamate receptor *N* ‐terminus demonstrate that divergence plays a dominant role in characterising the functions of VFTM [35]. Metabotropic glutamate receptor has longer *N* ‐terminus and is also found in earliest eukaryotes like chromalveolata, unikonts and opisthokonts [10].

**Fig. 3:**
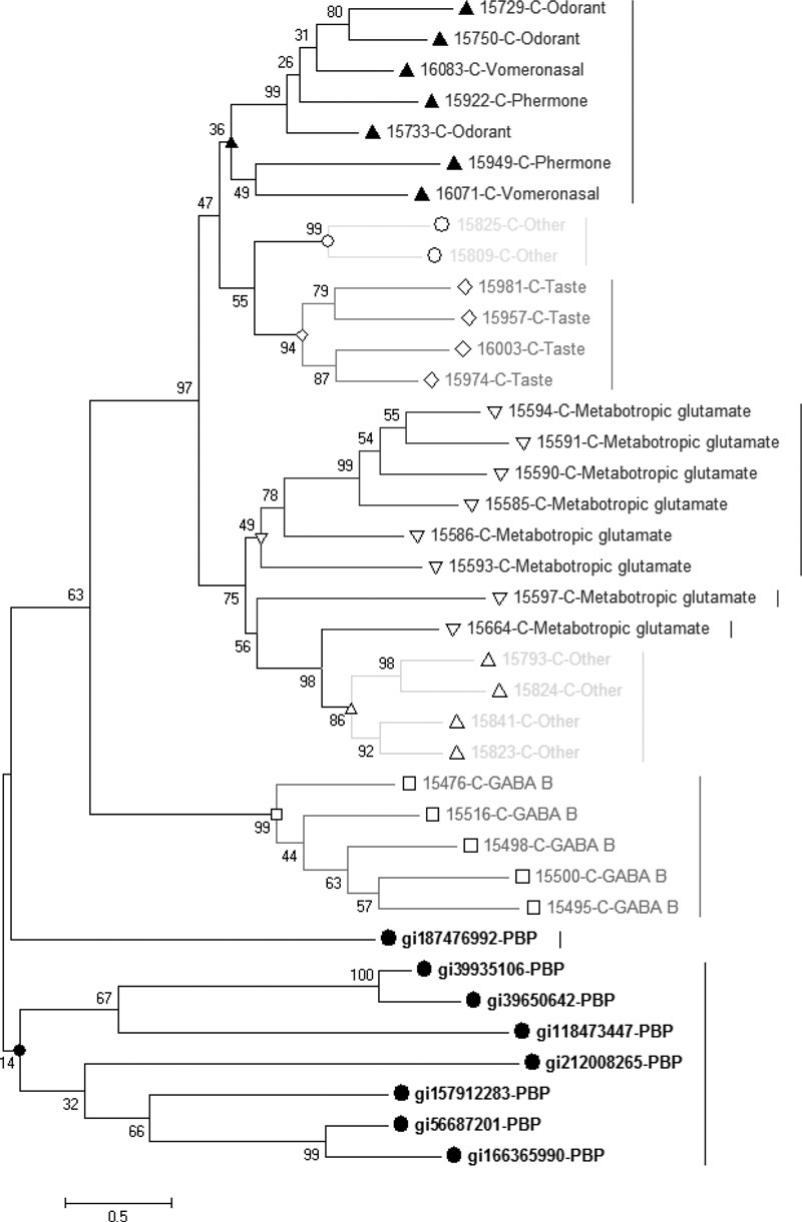
Phylogenetic tree of GPCR N‐terminus and PBP. (7‐transmembrane structure in the categories of prokaryote and eukaryote has an obvious disparity PBP have a closer sequence alignment result to GPCR subclass C, GABA_B, rather than the rest of GPCR. Abbreviations are in Fig. 1)

Sensitive sequence analysis techniques indicated that extracellular region of metabotropic glutamate receptor family is similar to PBP, but it failed to mention which subclass of metabotropic glutamate receptors is closer to PBP [36]. We extracted all GPCR *N* ‐terminus and preformed the comparative analysis between *N* ‐terminus and PBP. BLAST (*E* ‐value 0.01) result shows that only *N* ‐terminus from metabotropic glutamate receptor family could hit PBP sequences. However, *N* ‐terminus from other GPCR categories like secretin receptors and rhodopsin‐like receptor would not be hit even at such low standard (*E* ‐value 0.01). Therefore with the concern that *N* ‐terminus from metabotropic glutamate receptor family might be from PBP, we preformed phylogeny analysis (Fig. 3). The phylogenetic tree implies that GABA from metabotropic glutamate receptor family is much closer to PBP. Again, this indicates the extracellular domains of metabotropic glutamate receptor GABA_B might earlier evolve from PBP rather than other metabotropic receptors.

## 4 Conclusions

GPCR plays a key role in cellular signalling and probably evolved from prokaryotic world. Most rhodopsin‐like receptors only occur after metazoan and they would be the latest GPCR, which appeared after secretin receptor family and metabotropic glutamate receptor family. Highly conserved 7‐transmembrane region shares significant similarity with bacteriorhodopsin in prokaryotes. PBP would be the prototype of GPCR *N* ‐terminus. GABA from metabotropic glutamate receptor might be most ancient GPCR because both *N* ‐terminus and 7‐transmemebrane of GABA are closer to the ancient PBP and bacteriorhodopsin. Therefore it is possible that ancient PBP and bacteriorhodopsin combined via GPCR cysteine residue box and then formed the prototype of metabotropic glutamate receptor family.

**Fig. 4:**
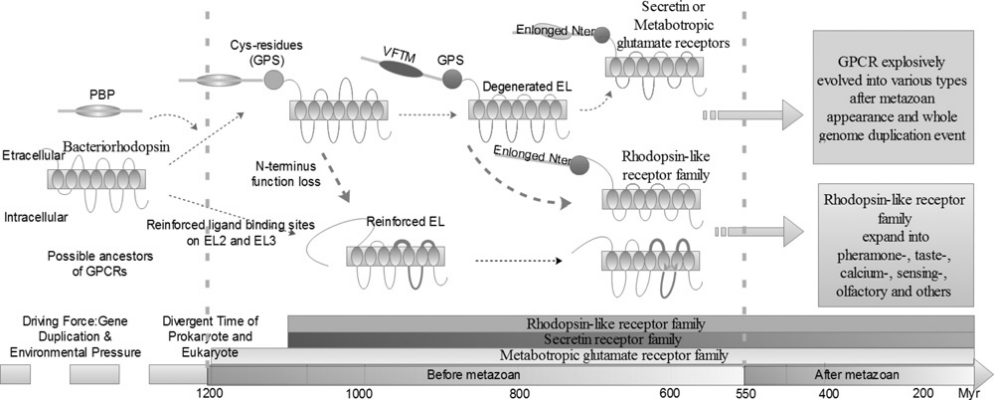
Overall presumption for GPCR evolutionary mechanism

Here our hypothesis is metabotropic glutamate receptor is the forefather of all GPCR and it probably evolves from the compound of PBP and bacteriorhodopsin (Fig. 4). Afterwards, with the enhancement of VFTM in *N* ‐terminus, it evolved into other secretin receptor family and metabotropic glutamate receptor family, as shown by black dash lines. As for rhodopsin‐like receptors, there would be two evolutionary mechanisms. One is that it would directly evolve from bacteriorhodopsin with reinforced ligand‐binding sites, which could take charge of receiving signals from extracellular instead of *N* ‐terminus (shown by blue dash line). The other is original metabotropic glutamate receptor lost the function of binding sites on *N* ‐terminus (shown by orange dash line) and therefore it must reinforce EL to bind signals. With reinforced binding function on EL, metabotropic glutamate receptor explosively expanded into more diversified subfamilies after the appearance of vertebrates.

## References

[1] Schioth H.B. , and Fredriksson R. : ‘The GRAFS classification system of G‐protein coupled receptors in comparative perspective’, Gen. Comparative Endocrinol., 2005, 142, (1–2), pp. 94–101 Epub 2005/05/03 (doi: 10.1016/j.ygcen.2004.12.018) 15862553

[2] Pin J.P. Galvez T. , and Prezeau L. : ‘Evolution, structure, and activation mechanism of family 3/C G‐protein‐coupled receptors’, Pharmacol. Ther., 2003, 98, (3), pp. 325–354. Epub 2003/06/05 (doi: 10.1016/S0163-7258(03)00038-X) 12782243

[3] Grigorieff N. Ceska T.A. Downing K.H. Baldwin J.M. , and Henderson R. : ‘Electron‐crystallographic refinement of the structure of bacteriorhodopsin’, J. Mol. Biol., 1996, 259, (3), pp. 393–421. Epub 1996/06/14 (doi: 10.1006/jmbi.1996.0328) 8676377

[4] Graul R.C. , and Sadee W. : ‘Evolutionary relationships among G protein‐coupled receptors using a clustered database approach’, AAPS PharmSci., 2001, 3, (2), pp. E12. Epub 2001/12/14 (doi: 10.1208/ps030212) 11741263 PMC2779559

[5] Fredriksson R. , and Schioth H.B. : ‘The repertoire of G‐protein‐coupled receptors in fully sequenced genomes’, Mol. Pharmacol., 2005, 67, (5), pp. 1414–1425. Epub 2005/02/03 (doi: 10.1124/mol.104.009001) 15687224

[6] Gloriam D.E. Bjarnadottir T.K. Yan Y.L. Postlethwait J.H. Schioth H.B. , and Fredriksson R. : ‘The repertoire of trace amine G‐protein‐coupled receptors: large expansion in zebrafish’, Mol. Phylogenet. Evol., 2005, 35, (2), pp. 470–482. Epub 2005/04/05 (doi: 10.1016/j.ympev.2004.12.003) 15804416

[7] Cardoso J.C. Pinto V.C. Vieira F.A. Clark M.S. , and Power D.M. : ‘Evolution of secretin family GPCR members in the metazoa’, BMC Evol. Biol., 2006, 6, pp. 108. Epub 2006/12/15 (doi: 10.1186/1471-2148-6-108) 17166275 PMC1764030

[8] Churcher A.M. , and Taylor J.S. : ‘The antiquity of chordate odorant receptors is revealed by the discovery of orthologs in the cnidarian Nematostella vectensis’, Genome Biol Evol., 2011, 3, pp. 36–43. Epub 2010/12/03 (doi: 10.1093/gbe/evq079) 21123836 PMC3017388

[9] Kurtenbach S. Mayer C. Pelz T. Hatt H. Leese F. , and Neuhaus E.M. : ‘Molecular evolution of a chordate specific family of G protein‐coupled receptors’, BMC Evol Biol., 2011, 11, pp. 234. Epub 2011/08/11 (doi: 10.1186/1471-2148-11-234) 21827690 PMC3238225

[10] Krishnan A. Almen M.S. Fredriksson R. , and Schioth H.B. : ‘The origin of GPCRs: identification of mammalian like Rhodopsin, Adhesion, Glutamate and Frizzled GPCRs in fungi’, PLoS One, 2012, 7, (1), pp. e29817. Epub 2012/01/13 (doi: 10.1371/journal.pone.0029817) 22238661 PMC3251606

[11] Nordstrom K.J. Sallman Almen M. Edstam M.M. Fredriksson R. , and Schioth H.B. : ‘Independent HHsearch, Needleman – Wunsch‐based, and motif analyses reveal the overall hierarchy for most of the G protein‐coupled receptor families’, Mol. Biol. Evol., 2011, 28, (9), pp. 2471–2480. Epub 2011/03/16 (doi: 10.1093/molbev/msr061) 21402729

[12] Li W. , and Godzik A. : ‘Cd‐hit: a fast program for clustering and comparing large sets of protein or nucleotide sequences’, Bioinformatics, 2006, 22, (13), pp. 1658–1659. Epub 2006/05/30 (doi: 10.1093/bioinformatics/btl158) 16731699

[13] Altschul S.F. Gish W. Miller W. Myers E.W. , and Lipman D.J. : ‘Basic local alignment search tool’, J. Mol. Biol., 1990, 215, (3), pp. 403–410. Epub 1990/10/05 2231712 10.1016/S0022-2836(05)80360-2

[14] Thompson J.D. Gibson T.J. , and Higgins D.G. : ‘Multiple sequence alignment using ClustalW and ClustalX’, Curr. Protoc. Bioinf., 2002, Chapter 2:Unit 2 3. Epub 2008/09/17 10.1002/0471250953.bi0203s00 18792934

[15] Yang Z. : ‘Maximum‐likelihood estimation of phylogeny from DNA sequences when substitution rates differ over sites’, Mol. Biol. Evol., 1993, 10, (6), pp. 1396–1401. Epub 1993/11/01 8277861 10.1093/oxfordjournals.molbev.a040082

[16] Tamura K. Peterson D. Peterson N. Stecher G. Nei M. , and Kumar S. : ‘MEGA5: molecular evolutionary genetics analysis using maximum likelihood, evolutionary distance, and maximum parsimony methods’, Mol. Biol. Evol., 2011, 28, (10), pp. 2731–2739. Epub 2011/05/07 (doi: 10.1093/molbev/msr121) 21546353 PMC3203626

[17] Felder C.B. Graul R.C. Lee A.Y. Merkle H.P. , and Sadee W. : ‘The Venus flytrap of periplasmic binding proteins: an ancient protein module present in multiple drug receptors’, AAPS PharmSci., 1999, 1, (2), pp. E2. Epub 2001/12/14 (doi: 10.1208/ps010202) 11741199 PMC2761117

[18] Fredriksson R. Gloriam D.E. Hoglund P.J. Lagerstrom M.C. , and Schioth H.B. : ‘There exist at least 30 human G‐protein‐coupled receptors with long Ser/Thr‐rich *N* ‐termini’, Biochem. Biophys. Res. Commun., 2003, 301, (3), pp. 725–734. Epub 2003/02/05 (doi: 10.1016/S0006-291X(03)00026-3) 12565841

[19] Vassilatis D.K. Hohmann J.G. Zeng H. Li F. Ranchalis J.E. , and Mortrud M.T. *et al*.: ‘The G protein‐coupled receptor repertoires of human and mouse’. Proc. Natl Acad. Sci. USA, 2003, 100, (8), pp. 4903–4098. Epub 2003/04/08 (doi: 10.1073/pnas.0230374100) 12679517 PMC153653

[20] Kristiansen K. : ‘Molecular mechanisms of ligand binding, signaling, and regulation within the superfamily of G‐protein‐coupled receptors: molecular modeling and mutagenesis approaches to receptor structure and function’, Pharmacol Ther., 2004, 103, (1), pp. 21–80. Epub 2004/07/15 (doi: 10.1016/j.pharmthera.2004.05.002) 15251227

[21] Trumpp‐Kallmeyer S. Hoflack J. Bruinvels A. , and Hibert M. : ‘Modeling of G‐protein‐coupled receptors: application to dopamine, adrenaline, serotonin, acetylcholine, and mammalian opsin receptors’, J Med Chem., 1992, 35, (19), pp. 3448–3462. Epub 1992/09/18 (doi: 10.1021/jm00097a002) 1328638

[22] Palczewski K. Kumasaka T. Hori T. Behnke C.A. Motoshima H. , and Fox B.A. *et al*.: ‘Crystal structure of rhodopsin: a G protein‐coupled receptor’, Science, 2000, 289, (5480), pp. 739–745. Epub 2000/08/05 (doi: 10.1126/science.289.5480.739) 10926528

[23] Oesterhelt D. : ‘The structure and mechanism of the family of retinal proteins from halophilic archaea’, Curr. Opin. Struct. Biol., 1998, 8, (4), pp. 489–500. Epub 1998/09/08 (doi: 10.1016/S0959-440X(98)80128-0) 9729742

[24] Fuhrman J.A. Schwalbach M.S. , and Stingl U. : ‘Proteorhodopsins: an array of physiological roles?’, Nat. Rev. Microbiol., 2008, 6, (6), pp. 488–494. Epub 2008/05/14 18475306 10.1038/nrmicro1893

[25] Zhang D. , and Weinstein H. : ‘Polarity conserved positions in transmembrane domains of G‐protein coupled receptors and bacteriorhodopsin’, FEBS lett., 1994, 337, (2), pp. 207–212. Epub 1994/01/10 (doi: 10.1016/0014-5793(94)80274-2) 8287978

[26] Fridmanis D. Fredriksson R. Kapa I. Schioth H.B. , and Klovins J. : ‘Formation of new genes explains lower intron density in mammalian Rhodopsin G protein‐coupled receptors’, Mol. Phylogenet. Evol., 2007, 43, (3), pp. 864–880. Epub 2006/12/26 (doi: 10.1016/j.ympev.2006.11.007) 17188520

[27] Sudo Y. Ihara K. Kobayashi S. Suzuki D. Irieda H. , and Kikukawa T. *et al*.: ‘A microbial rhodopsin with a unique retinal composition shows both sensory rhodopsin II and bacteriorhodopsin‐like properties’, J. Biol. Chem., 2011, 286, (8), pp. 5967–5976. Epub 2010/12/08 (doi: 10.1074/jbc.M110.190058) 21135094 PMC3057805

[28] Gross R. Wolf M.M. Schumann C. Friedman N. Sheves M. , and Li L. *et al*.: ‘Primary photoinduced protein response in bacteriorhodopsin and sensory rhodopsin II’, J. Am. Chem. Soc., 2009, 131, (41), pp. 14868–14878. Epub 2009/09/26 (doi: 10.1021/ja904218n) 19778046

[29] Klare J.P. Bordignon E. Engelhard M. , and Steinhoff H.J. : ‘Sensory rhodopsin II and bacteriorhodopsin: light activated helix F movement’, Photochem. Photobiol. Sci., 2004, 3, (6), pp. 543–547. Epub 2004/06/02 (doi: 10.1039/b402656j) 15170483

[30] Mizobe T. Maze M. Lam V. Suryanarayana S. , and Kobilka B.K. : ‘Arrangement of transmembrane domains in adrenergic receptors. Similarity to bacteriorhodopsin’, J. Biol. Chem., 1996, 271, (5), pp. 2387–2389. Epub 1996/02/02 (doi: 10.1074/jbc.271.5.2387) 8576196

[31] Pardo L. Ballesteros J.A. Osman R. , and Weinstein H. : ‘On the use of the transmembrane domain of bacteriorhodopsin as a template for modeling the three‐dimensional structure of guanine nucleotide‐binding regulatory protein‐coupled receptors’, Proc. Natl. Acad. Sci. USA, 1992, 89, (9), pp. 4009–4012. Epub 1992/05/01 (doi: 10.1073/pnas.89.9.4009) 1315046 PMC525621

[32] Schram V. , and Thompson T.E. : ‘Influence of the intrinsic membrane protein bacteriorhodopsin on gel‐phase domain topology in two‐component phase‐separated bilayers’, Biophys. J., 1997, 72, (5), pp. 2217–2225. Epub 1997/05/01 (doi: 10.1016/S0006-3495(97)78865-9) 9129824 PMC1184416

[33] Kawaguchi S. Kambara O. Shibata M. Kandori H. , and Tominaga K. : ‘Low‐frequency dynamics of bacteriorhodopsin studied by terahertz time‐domain spectroscopy’, Phys. Chem. Chem. Phys. PCCP., 2010, 12, (35), pp. 10255–10262. Epub 2010/07/08 (doi: 10.1039/b927397b) 20607183

[34] Taylor E.W. , and Agarwal A. : ‘Sequence homology between bacteriorhodopsin and G‐protein coupled receptors: exon shuffling or evolution by duplication?’, FEBS Lett., 1993, 325, (3), pp. 161–166. Epub 1993/07/05 (doi: 10.1016/0014-5793(93)81065-8) 8319802

[35] Cao J. Huang S. Qian J. Huang J. Jin L. , and Su Z. *et al*.: ‘Evolution of the class C GPCR Venus flytrap modules involved positive selected functional divergence’, BMC Evol. Biol., 2009, 9, pp. 67. Epub 2009/03/28 (doi: 10.1186/1471-2148-9-67) 19323848 PMC2670285

[36] O'Hara P.J. Sheppard P.O. Thogersen H. Venezia D. Haldeman B.A. , and McGrane V. *et al*.: ‘The ligand‐binding domain in metabotropic glutamate receptors is related to bacterial periplasmic binding proteins’, Neuron, 1993, 11, (1), pp. 41–52. Epub 1993/07/01 (doi: 10.1016/0896-6273(93)90269-W) 8338667

